# Reduced recurrence of atrial fibrillation after cryoballoon ablation in patients with preprocedural patent foramen ovale: a retrospective single-center observational cohort study

**DOI:** 10.3389/fcvm.2026.1840929

**Published:** 2026-06-30

**Authors:** Stefano Bonapace, Alessandro Costa, Alessio Marinelli, Laura Lanzoni, Giulio Molon

**Affiliations:** 1 Department of Cardiology, IRCCS Sacro Cuore – Don Calabria, Negrar di Valpolicella, Italy; 2 Department of Cardiology, AOOR Villa Sofia-Cervello, Palermo, Italy

**Keywords:** atrial fibrillation, atrial septal aneurysm, cryoballoon ablation, patent foramen ovale, transoesophageal echocardiography

## Abstract

**Background:**

Atrial septal anomalies such as patent foramen ovale (PFO), atrial septal aneurysm (ASA), and lipomatous hypertrophy of the interatrial septum (LHIS) are frequently identified by transoesophageal echocardiography (TEE) in patients undergoing atrial fibrillation (AF) ablation. While ASAs have been linked to arrhythmogenic potential, their influence on post-ablation outcomes, particularly after cryoballoon ablation (CBA), is poorly understood.

**Methods:**

In this retrospective single-center cohort study we enrolled all patients who underwent CBA-based pulmonary vein isolation (PVI) for paroxysmal or persistent AF between June 2015 and April 2024 at our center and had preprocedural TEE imaging. Interatrial septal morphology—including PFO, ASA, and LHIS—was assessed. The primary endpoint was AF recurrence, defined as any episode lasting >30 s beyond the 90-day blanking period. Kaplan–Meier analysis and Cox proportional hazards modeling were used for time-to-event analyses.

**Results:**

Among 657 patients (mean age 61.7 ± 9.6 years; 71.2% male), PFO was identified in 16.6%, LHIS in 5.7%, and ASA in 1.0%. Over a median follow-up of 19 months (IQR: 3–76), AF recurrence occurred in 26.8%. Kaplan–Meier analysis revealed a significantly lower recurrence rate in patients with PFO compared to those without PFO (HR: 0.62; 95% CI: 0.43–0.89; *p* = 0.010). This association was independent from other clinical, electrophysiological and echocardiographic confounders (HR: 0.56; 95% CI: 0.38–0.83; *p* = 0.004).

**Conclusions:**

In this single-center cohort, the presence of PFO was independently associated with a lower risk of AF recurrence following CBA. These findings suggest a potential protective role of PFO and merit further prospective investigation.

## Graphical Abstract

**Figure:**
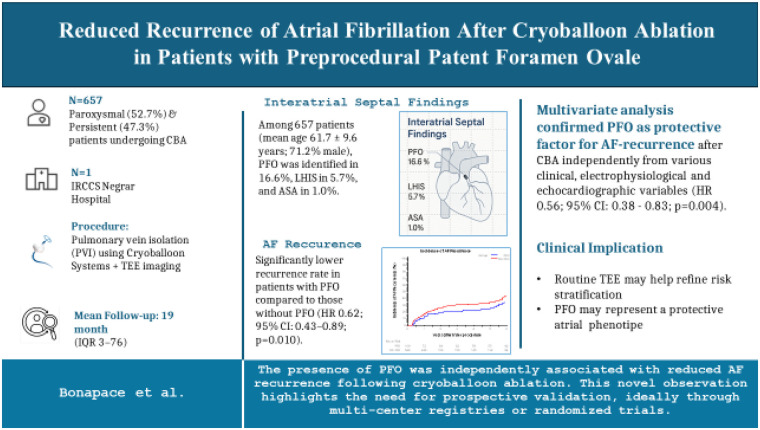

## Introduction

Atrial septal abnormalities, including patent foramen ovale (PFO), atrial septal aneurysm (ASA), lipomatous hypertrophy of the interatrial septum (LHIS) are not rarely found in patients undergoing transesophageal echocardiography (TEE) prior to atrial fibrillation (AF) ablation (1). These anomalies, particularly ASA and the combination of ASA with PFO, have been associated with atrial arrhythmias and are thought to reflect an underlying atrial myopathy, which may increase atrial vulnerability by modifying the electrophysiological substrate and promoting paroxysmal AF (2–8). However, there is a lack of data in the literature regarding the possible relationship between atrial septal anomalies and AF recurrence following cryoballoon ablation (CBA). To date, only one group of authors reported a possible independent association between PFO and AF recurrence following CBA (9, 10). In this contest, we retrospectively evaluated interatrial septal morphology and characteristics assessed by TEE in all consecutive patients who underwent CBA at our center between 2015 and 2024. Our aim was to investigate whether TEE-derived IAS parameters may serve as prognostic markers of AF recurrence after CBA procedure.

## Methods

This was a retrospective, single-center analysis including all consecutive patients who underwent pulmonary vein isolation (PVI) using a CBA-based ablation technique for the treatment of recurrent AF—both paroxysmal and persistent—between June 2015 and April 2024. All procedures were performed according to international consensus recommendations, which endorse PVI as the cornerstone of catheter ablation for patients with both paroxysmal and persistent AF (11). Patients were included if a TEE was available within 30 days prior to the ablation procedure, allowing detailed assessment of IAS morphology. This analysis was conducted as part of the 1STOP project, a national, cardiovascular data repository and medical care quality initiative developed within the One Hospital ClinicalService (OHCS) framework. The 1STOP project specifically focuses on the management of AF patients treated with AF ablation in routine Italian clinical practice and aims to promote data-driven optimization of clinical workflows and procedural outcomes. Procedures and data collection were carried out in accordance with the ethical principles of the Declaration of Helsinki and received approval by the local institutional review board of Verona and Rovigo. Written informed consent was obtained from all participants enrolled in the One Hospital Clinical Service project.

### Data collection

Baseline demographic, clinical, and procedural data were collected during the baseline visit, the ablation procedure, and follow-up evaluations. At baseline, a comprehensive clinical assessment was performed, including patient medical history, comorbidities, duration of atrial fibrillation, and AF-related symptoms. Follow-up data were obtained through scheduled outpatient visits and included. AF recurrence was defined as any documented episode of AF lasting more than 30 s, occurring beyond a standard 90-day post-ablation blanking period. Atrial fibrillation had to be documented by ECG, Holter monitoring, or from the implanted loop recorder. Moreover, all patients were advised to obtain an ECG if they experienced palpitations.

### Transesophageal echocardiography

A standardized TEE was conducted in all patients prior to the procedure to exclude the presence of left atrial and left auricular thrombus. Specific TEE-derived interatrial septum features—including PFO, ASA, and lipomatous hypertrophy of the interatrial septum (LHIS)—were assessed and documented by an experienced echocardiographer during the baseline evaluation. PFO was defined as the presence of a communication between the septum primum and septum secundum with color Doppler-detected left-to-right shunt or as a right-to-left contrast passage on bubble study (12). ASA was deﬁned according to criteria previously published (4, 5, 13): 1) diameter of the base of the aneurismal portion of the IAS 15 mm or more and either 2) protrusion of the IAS, or part of it, 15 mm or more beyond the plane of the IAS or 3) phasic excursion of the IAS during the cardiorespiratory cycle 15 mm or more in total amplitude. Lipomatous hypertrophy of the interatrial septum was defined by TEE as a dumbbell-shaped, hyperechogenic thickening (>20 mm) of the interatrial septum sparing the fossa ovalis (7, 8, 14, 15).

### Statistical analysis

Descriptive statistics were used to summarize all variables. Continuous variables are reported as mean ± standard deviation, median with interquartile range (IQR), as well as minimum and maximum values, where appropriate. Categorical variables are presented as absolute counts and percentages. For time-to-event analyses of AF recurrence, the Kaplan–Meier method was used to estimate cumulative incidence rates, with corresponding 95% confidence intervals (CIs). The last contact date was defined as the latest among in-hospital follow-up visits, telephone contacts, clinical event dates, or formal exit dates, whichever occurred most recently. To evaluate the association between AF recurrence and baseline clinical or echocardiographic characteristics, univariate and multivariate Cox proportional hazards models were applied. Hazard ratios (HRs) with 95% CIs were reported to quantify relative risks. The proportional hazards assumption was formally tested for all models. For multivariable modelling, a stepwise variable selection approach was used, with a liberal entry criterion of *p* < 0.30 and a retention criterion of *p* < 0.10, to identify independent predictors of AF recurrence, with a significance level set at *p* < 0.05. In addition, a fully adjusted multivariable Cox regression model was performed including clinically relevant covariates known to be associated with AF recurrence after catheter ablation. To reduce the risk of model overfitting, variables with very low prevalence in the study population were not included in the final model. Collinearity between candidate variables was assessed before model construction, and collinear variables were excluded from the final multivariable analysis. All analyses were conducted using SAS software, version 9.4 (SAS Institute Inc., Cary, NC, USA).

## Results

As shown in Table 1 a total of 657 patients was included (mean age 61.7 ± 9.6 years; 71.2% male). Paroxysmal AF was present in 52.7%, and persistent AF in 47.3%. Median time from first AF episode was 25 months (IQR: 12–58). PFO was present in 16.6%, LHIS in 5.7%, and ASA in 1.0% of patients. LA size was normal in 46.5%, mildly dilated in 30.8%, moderately dilated in 13.8%, and severely dilated in 8.8%. Mitral regurgitation was mostly mild (92.0%). No significant differences in baseline clinical characteristics were observed between patients with PFO and those without PFO. The only echocardiographic significant differences were related to atrial septal anatomical features, including the prevalence of PFO, septal aneurysm and overlapping septal abnormalities. Left atrial dimensions and mitral regurgitation were not significantly different between the two groups.

**Table 1:** Baseline clinical and echocardiographic characteristics in the whole population and in those with and without PFO.

Baseline characteristics	TOTAL (*N* = 657)	PFO (*N* = 109)	No PFO (*N* = 548)	*p*-value
Age at first cryoablation (yrs) (Mean ± SD)	61.7 ± 9.6	61.8 ± 8.9	61.6 ± 9.8	0.828
Gender (Male)	71.2% (468)	67.9% (74)	71.9% (394)	0.399
BMI (Kg/m^2^) (Mean ± SD)	26.2 ± 4.1	26.3 ± 3.8	26.2 ± 4.1	0.566
Type of Atrial fibrillation				0.119
Paroxysmal	52.7% (346)	44.0% (48/109)	54.4% (298/548)	
Persistent	40.6% (267)	46.8% (51/109)	39.4% (216/548)	
Long Standing Persistent	6.7% (44)	9.2% (10/109)	6.2% (34/548)	
Months from first AF Episode, median (IQR)	25.0 (12.0–58.0)	35.0 (11.0–60.0)	25.0 (12.0–55.0)	0.969
NYHA class ≥2	14.6% (96)	15.0% (17)	13.6% (79)	0.841
History of Stroke/transient Ischemic attack	4.1% (27)	1.9% (2)	4.6% (25)	0.225
Hypertension	61.5% (404)	61.4% (67)	61.5% (337)	0.896
Diabetes	9.3% (61)	9.1% (10)	9.3% (51)	0.953
Kidney Disease	1.4% (9)	0% (0)	1.8% (9)	0.195
CHA_2_DS_2_-VASc				0.964
0	20.1% (132)	20.2% (22)	20.1% (110)	
1	32.3% (212)	33.0% (36)	32.1% (176)	
2	27.2% (179)	28.4% (31)	27.0% (148)	
3	14.3% (94)	11.9% (13)	14.8% (81)	
4	4.7% (31)	5.5% (6)	4.6% (25)	
≥5	1.4% (9)	0.9% (1)	1.5% (8)	
Drug Therapy				
Thrombin Inhibitor	23.0% (151)	25.7% (28)	22.4% (123/)	0.462
Factor Xa Inhibitor	33.5% (220)	24.8% (27)	35.2% (193)	0.035
Class III or Class I AAD	59.8% (393)	62.4% (68)	59.3% (325)	0.549
Class I or II or III AAD	81.4% (535)	80.7% (88)	81.6% (447)	0.838
ACE-inhibitors	38.7% (254)	37.6% (41)	38.9% (213)	0.806
Diuretics	13.4% (76)	9.7% (10)	14.3% (66)	0.221
Echo Parameters				
Left Ventricular Ejection Fraction (Mean ± SD)	56.9% ± 8.7	59.3 ± 8.1	56.2 ± 8.9	0.139
Lipomatous Atrial Septal Hypertrophy (LHIS)	5.7% (37)	3.7% (4)	6.2% (33)	0.306
Patent Foramen Ovale (PFO)	16.6% (109)	100% (109)	0% (0)	<0.001
Aneurysmal septum	1.0% (6)	4.6% (5)	0.2% (1)	<0.001
LHIS and PFO	0.7% (4)	3.7% (4)	0% (0)	<0.001
PFO and Aneurysmal septum	0.9% (5)	4.6% (5)	0% (0)	<0.001
LHIS and Aneurysmal septum	0% (0)	0% (0)	0% (0)	-
LHIS, PFO and Aneurysmal septum	0% (0)	0% (0)	0% (0)	-
Left atrium dimension				0.853
Normal	46.5% (306)	44.7% (49)	47.1% (257)	
Mildly dilated	30.8% (202)	34.2% (38)	29.8% (164)	
Moderately dilated	13.8% (91)	10.5% (11)	14.9% (80)	
Severely dilated	8.8% (58)	10.5% (11)	8.3% (47)	
Mitral valve insufficiency				0.744
No	2.9% (19)	1.9% (2)	3.2% (17)	
Mild	92.0% (605)	92.5% (101)	91.9% (507)	
Moderate	5.1% (33)	5.7% (6/106)	4.9% (27)	
Severe	0.0% (0)	0% (0)	0% (0)	

Data are presented as mean ± standard deviation for continuous variables or median (Interquartile Range (IQR) and percent (number) for categorical variables. AAD, Anti Arrhythmic Drug; BMI, Body Mass Index; PFO, patent foramen ovale; ASA, atrial septal aneurysm; LHIS, lipomatous hypertrophy of the interatrial septum; MI, mitral insufficiency; ACE inhibitors, Angiotensin-Converting Enzyme inhibitors.

Table 2 shows the procedural characteristics and complications. As reported the median procedure time was 60 min (IQR: 50–70), the fluoroscopy time was 16 min (IQR: 12–22), and the acute success rate was more than 98% for all pulmonary veins. No major complications (e.g., stroke, tamponade, and fistula) occurred. Minor complications included transient diaphragmatic paralysis (1.2%) and groin hematoma (0.3%).

**Table 2:** Procedural characteristics of cryoballoon ablation procedure and acute complications.

Procedural characteristics	Total (*N* = 657)
Procedure duration (min)	60.0 (50.0–70.0)
Fluoroscopy duration (min)	16.0 (12.0–22.0)
Left atrium dwell time (min)	35.0 (30.0–40.0)
Ablation time (min)	15.0 (12.0–30.0)
Acute Success Rate (Left Superior Pulmonary Vein)	99.7% (655)
Acute Success Rate (Left Inferior Pulmonary Vein)	99.5% (653)
Acute Success Rate (Right Superior Pulmonary Vein)	100.0% (657)
Acute Success Rate (Right Inferior Pulmonary Vein)	98.4% (646)
No mapping usage	100.0% (657)
Pre-ablation rhythm	
Synus	82.8% (544)
FA	17.2% (113)
Cardioversion	14.2% (93)
Post-ablation rhythm	
Synus	97.5% (640)
FA	2.5% (16)
Periprocedural Complications	
Patients with at least one acute periprocedural event	1.7% (1)
Permanent Diaphragmatic paralysis	0.0% ()
Transient Diaphragmatic Paralysis	1.2% (8)
Pericardial effusion	0.0% (0)
AV fistula	0.0% (0)
Cardiac tamponade	0.0% (0)
Pneumothorax/Haemothorax	0.0% (0)
Femoral artery pseudoaneurysm	0.2% (1)
Stroke a/o TIA	0.0% (0)
Pulmonary vein stenosis	0.0% (0)
Hematoma	0.3% (2)

Data are presented as median (interquartile range) for continuous variables and as number (%) for categorical variables. Acute complications were defined as any adverse event occurring during the index procedure or hospital stay.

**Figure 1:**
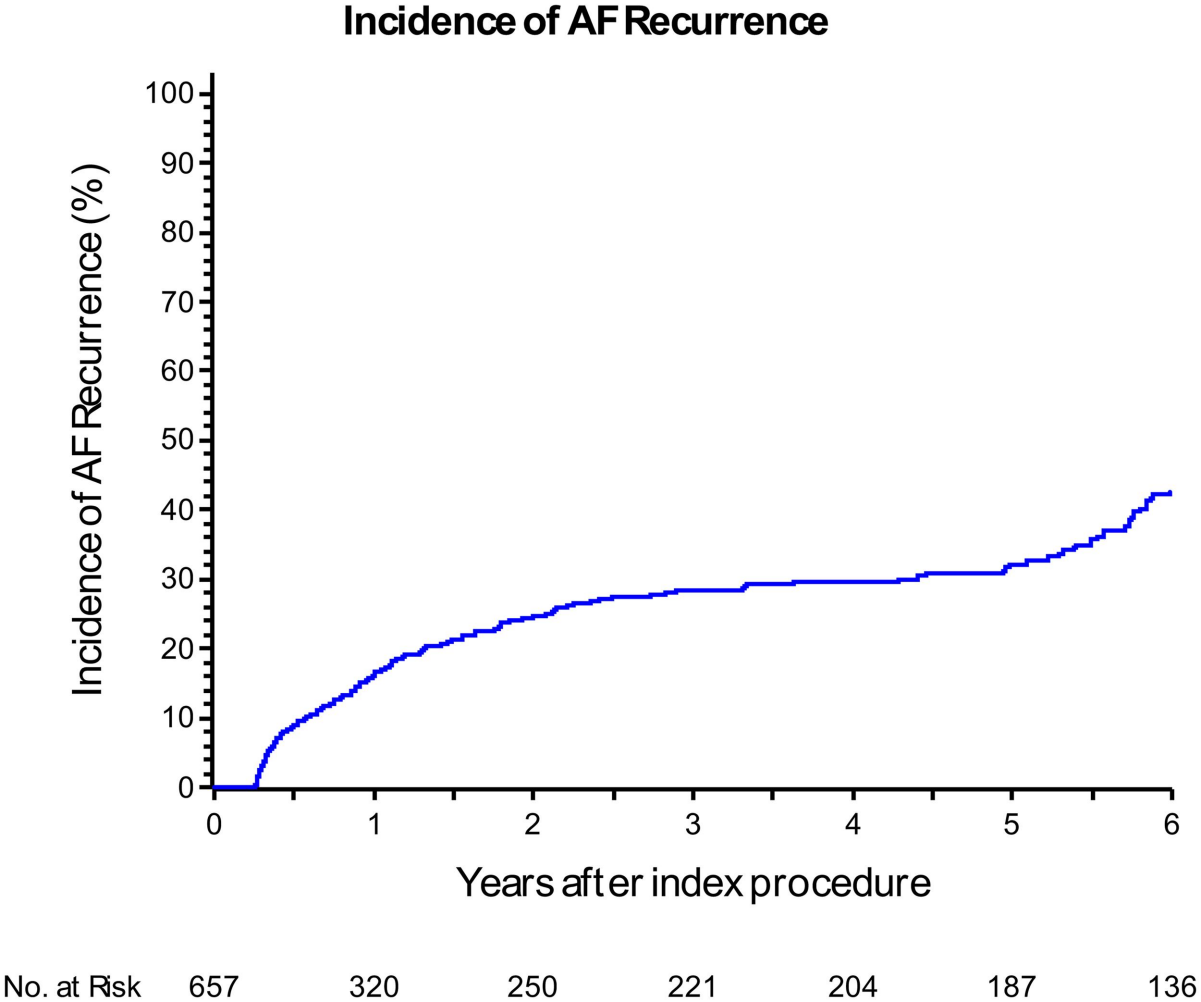
Kaplan–Meier curve showing the cumulative incidence of first atrial fibrillation (AF) recurrence after pulmonary vein isolation in the overall study population. The 1-year recurrence-incidence was 16.1% (95% CI: 12.9%–20.0%), increasing to 32.2% (95% CI: 27.7%–37.3%) at 5 years and 42.6% (95% CI: 37.3%–48.3%) at 6 years.

**Figure 2:**
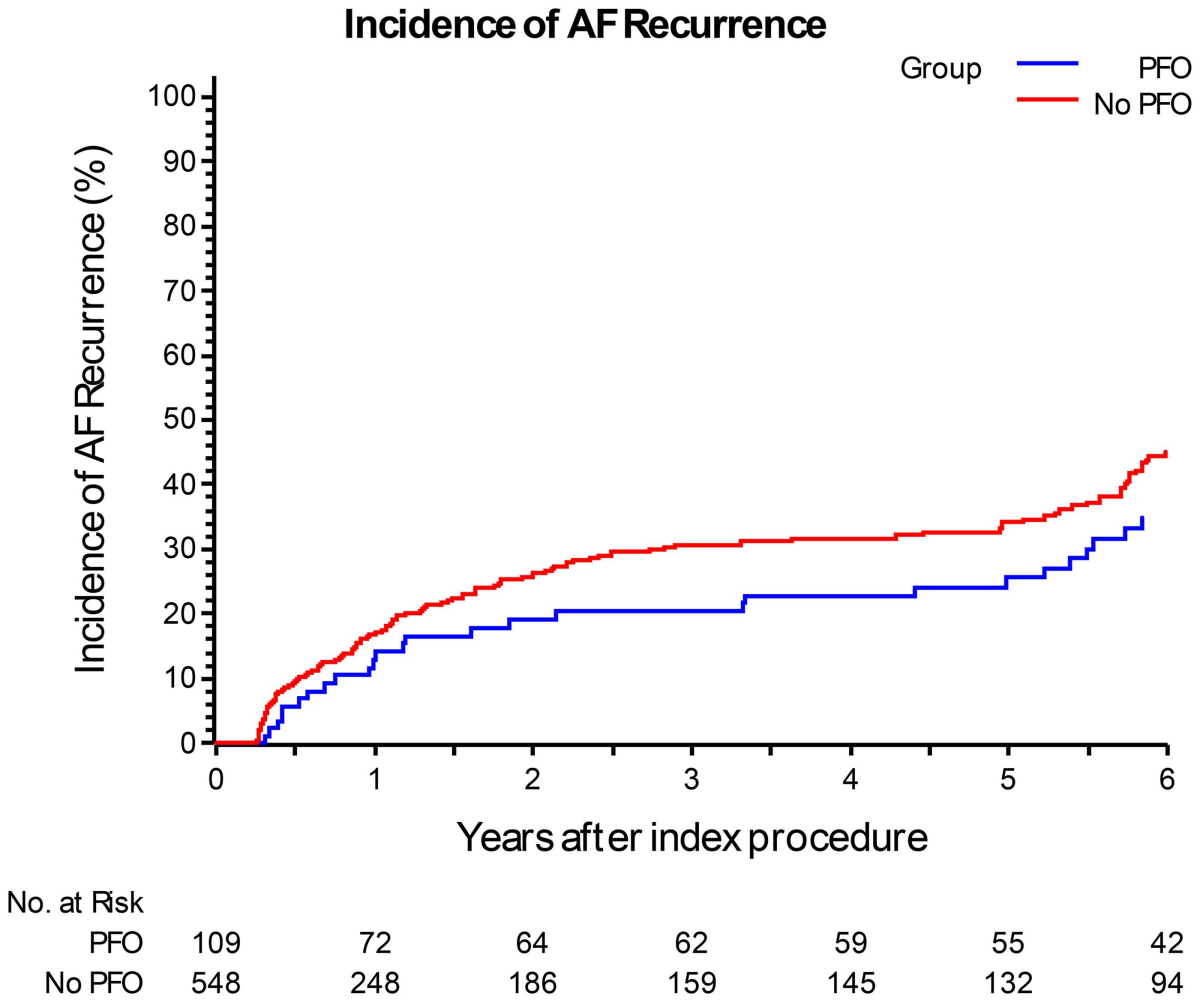
Kaplan–Meier analysis of time to first atrial fibrillation (AF) recurrence stratified by the presence or absence of a patent foramen ovale (PFO). AF recurrence occurred earlier and more frequently in patients without PFO. The hazard ratio for recurrence in the PFO group was 0.62 (95% CI: 0.43–0.89, *p* = 0.010).

At median follow-up of 19 months, 176 patients (26.8%) had AF recurrence. One-year recurrence-free survival was 83.9% (95% CI: 80–87), dropping to 67.8% (95% CI: 63–72) at 5 years (Figure 1). As detailed in Figure 2 stratified analysis showed recurrence in 25.5% of PFO patients vs. 31.8% without PFO (HR: 0.62; 95% CI: 0.43–0.89; *p* = 0.010). The incidence of recurrence remained consistently higher across timepoints in the subgroup without PFO compared to patients with PFO. As shown in Table 3 and in the Supplementary Table 1 multivariate analysis confirmed PFO as protective factor for AF-recurrence after CBA independently from various clinical, electrophysiological and echocardiographic variables (HR: 0.56; 95% CI: 0.38–0.83; *p* = 0.004).

**Table 3:** Univariate and multivariate stepwise Cox regression analysis for predictors of atrial fibrillation (AF) recurrence. The presence of a patent foramen ovale was independently associated with a lower risk of AF recurrence.

Univariate analysis			Multivariate analysis	
Variable	HR (95% CI)	*p*-value	HR (95% CI)	*p*-value
Age at first cryoablation (yrs) (continuous)	1.26 (0.95–1.67)	0.105	–	ns
Gender (Male)	1.22 (0.88–1.68)	0.234	–	ns
Persistent AF (vs. Paroxysmal AF)	1.01 (0.99–1.02)	0.416		
Cardiac Insufficiency	1.42 (0.58–3.47)	0.444		
Hypertension	1.08 (0.80–1.45)	0.625		
Diabetes	0.98 (0.54–1.76)	0.939		
Chronic kidney disease	1.91 (0.61–6.02)	0.267	–	ns
Procedure Duration (min)	1.00 (1.00–1.01)	0.204		
Overall average balloon temperature (Min, °C)	1.00 (0.97–1.03)	0.822		
Periprocedural Adverse Event	0.97 (0.43–2.19)	0.944		
LHIS	0.71 (0.29–1.72)	0.442		
PFO	0.62 (0.43–0.89)	0.010	0.56 (0.38–0.83)	0.004
ASA	0.45 (0.06–3.21)	0.425		
Normal LA	0.72 (0.44–1.19)	0.197		
Mildly dilated LA	0.79 (0.46–1.33)	0.367		
Moderately dilated LA	1.72 (0.95–3.11)	0.073		
Severely dilated LA	2.02 (0.96–4.26)	0.066		
No MI	0.15 (0.02–1.10)	0.063		
Mild MI	1.20 (0.71–2.05)	0.491		
Moderate MI	1.24 (0.72–2.14)	0.444		
Class I or III AAD	0.80 (0.60–1.06)	0.123	–	ns
Class I or II or III AAD	0.95 (0.65–1.38)	0.782		

HR, hazard ratio; CI, confidence interval; AF, atrial fibrillation; AAD, Anti Arrhythmic Drug; BMI, Body Mass Index; PFO, patent foramen ovale; ASA, atrial septal aneurysm; LHIS, lipomatous hypertrophy of the interatrial septum; MI, mitral insufficiency; ns, not significant. The multivariable analysis was performed using a stepwise selection procedure with a liberal entry criterion of *p* < 0.30 and a retention criterion of *p* < 0.10, to identify independent predictors of AF recurrence, with a significance level set at *p* < 0.05. Therefore, only the final model, including the variables retained after the selection process, is presented. – shows the variables entered in the model that were not retained.

## Discussion

Our findings suggest that the presence of a PFO may confer a significant protective effect against AF recurrence after cryoballoon ablation (CBA). Among 657 patients, PFO was identified in 16.6%, LHIS in 5.7%, and ASA in 1.0%. Over a median follow-up of 19 months, AF recurrence occurred in 26.8%, and Kaplan–Meier analysis revealed a lower recurrence rate in patients with PFO compared to those without PFO (*p* = 0.010). This observation warrants in-depth discussion regarding its potential pathophysiological underpinnings, clinical implications, and divergence from existing literature. Notably, our findings contrast with those of Kiełbasa et al., who reported increased AF recurrence in PFO patients undergoing CBA (9).

A central question is why a PFO might reduce the risk of recurrent AF after ablation. One hypothesis posits that left-to-right interatrial shunting through a PFO may decompress the left atrium, particularly under conditions of elevated atrial pressure or atrial remodeling. By attenuating atrial stretch and wall stress—key contributors to arrhythmogenesis—PFO may reduce the propensity for ectopic activity and re-entrant circuits (16, 17). This hypothesis is indirectly supported by studies of PFO closure, where elimination of the shunt has been associated with an increased incidence of new-onset AF, particularly in the early postprocedural phase (18–23).

However, it is speculable that the mechanisms underlying AF after PFO closure appear to be multifactorial and extend beyond simple hemodynamic changes. Mechanical factors, including device-related atrial irritation, local stretch, and altered septal compliance, may promote arrhythmogenesis, as highlighted in recent mechanistic reviews (24). In parallel, inflammatory responses induced by device implantation, endothelialization, and local tissue injury may transiently increase atrial vulnerability and trigger AF episodes (25). These data suggest that post-closure AF reflects a complex interplay between mechanical stress and inflammation rather than solely the loss of a decompressive shunt.

Importantly, the hemodynamic explanation for our findings requires careful interpretation. PFO is a congenital condition, and patients typically maintain relatively stable atrial pressures over time (26). Therefore, baseline interatrial shunting alone may not fully explain the reduced recurrence observed after CBA. An alternative possibility is that cryoballoon ablation itself induces transient changes in atrial compliance, pressure distribution, and autonomic tone (27–29). Acute post-ablation edema, inflammation, and stiffness causes significant rise in pulmonary artery and atrial pressures (29) which in patients with PFO could be partially mitigated by interatrial shunting. This interaction may be particularly relevant in the early post-ablation period (27–29) and could contribute to the observed differences in recurrence.

Beyond hemodynamics, the anatomical features of a PFO may exert electrical effects. The flap-like structure between the septum primum and secundum could act as a partial barrier to macro-reentrant circuits, altering conduction pathways across the interatrial septum and modulating arrhythmogenicity.

At the same time, an opposing hypothesis must be acknowledged. Atrial septal abnormalities, including PFO and ASA, have been associated with increased atrial vulnerability and a potentially arrhythmogenic substrate (3, 6). Early evidence suggested that interatrial septal abnormalities may be linked to atrial conduction disturbances and embolic risk, possibly reflecting an underlying atrial cardiomyopathy rather than a benign anatomical variant (3). From this perspective, PFO could represent a marker of intrinsic atrial disease predisposing to AF, rather than a protective factor. The coexistence of these competing mechanisms underscores the complexity of the relationship between septal anatomy and arrhythmogenesis and supports a more nuanced interpretation of our findings.

The discrepancy between our findings and those reported by Kiełbasa et al. (9) deserves further consideration. Several factors may account for these divergent results. Differences in patient populations—including age, AF type, and comorbidity burden—may influence both atrial substrate and the functional impact of PFO. Variability in PFO characterization (e.g., shunt size, associated ASA), imaging techniques, and diagnostic criteria may also contribute. Procedural factors, such as ablation protocol, operator experience, and peri-procedural management, may further modulate outcomes. Additionally, differences in follow-up intensity and rhythm monitoring strategies could influence AF detection rates. It is also plausible that PFO exerts a context-dependent effect, being protective in certain hemodynamic or structural conditions while detrimental in others.

Epidemiological considerations should also be taken into account. PFO is more prevalent in younger individuals (24), who generally exhibit a more favorable atrial substrate and lower AF burden (30). Thus, the observed association between PFO and reduced recurrence may partly reflect residual confounding, despite multivariable adjustment.

The pathophysiological interplay between PFO, atrial remodeling, inflammation, and neurohumoral activation warrants further investigation. Advanced imaging modalities such as cardiac MRI and 4D flow analysis, along with invasive hemodynamic studies, may help clarify how small interatrial shunting influences atrial mechanics and electrophysiology. These findings also have implications for the growing overlap between electrophysiology and structural heart interventions. As more patients undergo both PFO closure and AF ablation, the timing and sequencing of these procedures become clinically relevant. Closure of a PFO prior to ablation could eliminate a compensatory mechanism or alter atrial mechanics, potentially influencing ablation outcomes.

From a clinical perspective, these findings raise important considerations. Whether PFO should be regarded as a protective marker in selecting patients for AF ablation remains uncertain. Routine screening for PFO is not currently recommended unless clinically indicated; however, if confirmed in prospective studies, assessment of septal anatomy could contribute to improved risk stratification. It also remains unclear whether this potential protective effect is specific to CBA or extends to other ablation modalities, such as radiofrequency or pulsed field ablation.

### Limitations

This retrospective single-center design limits generalizability. Asymptomatic arrhythmia episodes may have been missed due to intermittent monitoring, except in patients with implantable loop recorders. PFO diagnosis via TEE, although standard, may underestimate true prevalence compared to transseptal catheterization or intracardiac echocardiography (10, 31, 32). Another limitation of the present study is that left atrial size was available only as a categorical echocardiographic classification because transesophageal echocardiography does not allow a precise calculation of left atrial volume Therefore, left atrial size could not be analyzed as a continuous variable, potentially reducing statistical granularity and power. Moreover, the number of patients with ASA and LHIS was small, limiting statistical power to detect their effects. Residual confounding cannot be excluded despite multivariable adjustment

## Conclusions

The presence of PFO was independently associated with reduced AF recurrence following cryoballoon ablation. This novel observation highlights the need for prospective validation, ideally through multi-center registries or randomized trials. Further mechanistic research is also warranted to determine whether PFO is a protective substrate or a surrogate marker for favourable atrial phenotype.

## Data Availability

The original contributions presented in the study are included in the article/Supplementary Material, further inquiries can be directed to the corresponding author.
