# Supplementary material for: Reduced recurrence of atrial fibrillation after cryoballoon ablation in patients with preprocedural patent foramen ovale: a retrospective single-center observational cohort study
Source: Front Cardiovasc Med. 2026 Jun 30;13:1840929. doi: 10.3389/fcvm.2026.1840929 (PMC13364578; doi:10.3389/fcvm.2026.1840929)
Supplement: Supplementary file 2 [file Table1.docx]

**Supplementary Table 1:** Univariate and fully adjusted multivariate Cox regression analysis for predictors of atrial fibrillation (AF) recurrence. The presence of a patent foramen ovale was independently associated with a lower risk of AF recurrence.

| **Univariate Analysis** | |  | **Multivariate Analysis** | |
| --- | --- | --- | --- | --- |
| **Variable** | **HR (95% CI)** | **p-value** | **HR (95% CI)** | **p-value** |
| **Age at first cryoablation (yrs) (continuous)** | 1.26 (0.95 - 1.67) | 0.105 | 1.01 (0.99 - 1.03) | 0.303 |
| **Gender (Male)** | 1.22 (0.88 - 1.68) | 0.234 | 1.26 (0.83 - 1.92) | 0.278 |
| **Persistent AF (vs. Paroxysmal AF)** | 1.01 (0.99 - 1.02) | 0.416 | 1.10 (0.78 - 1.56) | 0.593 |
| **Cardiac Insufficiency** | 1.42 (0.58 - 3.47) | 0.444 | 2.24 (0.52 - 9.72) | 0.283 |
| **Hypertension** | 1.08 (0.80 - 1.45) | 0.625 | 1.05 (0.72 - 1.53) | 0.811 |
| **Procedure Duration (min)** | 1.00 (1.00 - 1.01) | 0.204 | 1.00 (1.00 - 1.01) | 0.149 |
| **Overall average balloon temperature (Min,°C)** | 1.00 (0.97 - 1.03) | 0.822 | 1.00 (0.97 - 1.03) | 0.822 |
| **Periprocedural Adverse Event** | 0.97 (0.43 - 2.19) | 0.944 | 1.22 (0.49 - 3.06) | 0.668 |
| **LASH** | 0.71 (0.29 - 1.72) | 0.442 | 1.23 (0.44 - 3.46) | 0.693 |
| **PFO** | 0.62 (0.43 - 0.89) | 0.010 | 0.56 (0.38 - 0.83) | 0.004 |
| **ASA** | 0.45 (0.06 - 3.21) | 0.425 | 0.52 (0.07 - 3.91) | 0.526 |
| **Mild MI** | 1.20 (0.71 - 2.05) | 0.491 | 3.45 (0.45 - 26.68) | 0.235 |
| **Moderate MI** | 1.24 (0.72 - 2.14) | 0.444 | 4.42 (0.52 - 37.39) | 0.172 |
| **Class I or II or III AAD** | 0.95 (0.65 - 1.38) | 0.782 | 0.97 (0.59 - 1.59) | 0.897 |

HR = hazard ratio; CI = confidence interval; AF: atrial fibrillation; AAD: Anti Arrhythmic Drug; BMI= Body Mass Index; PFO = patent foramen ovale; ASA: atrial septal aneurysm: LHIS: lipomatous hypertrophy of the interatrial septum. MI: mitral insufficiency.

To reduce the risk of model overfitting, variables with very low prevalence in the study population were not included in the fully adjusted multivariable model. In addition, collinearity between candidate variables was assessed before model construction. Fisher’s exact test demonstrated a significant association between persistent AF and left atrial dimension (p = 0.045); therefore, left atrial dimension was excluded from the fully adjusted multivariable model because of collinearity with AF type.
